# Biological sensitivity to environmental context fluctuates dynamically within individuals from day to day

**DOI:** 10.1038/s41598-022-14481-7

**Published:** 2022-07-01

**Authors:** Emma Armstrong-Carter, Eva H. Telzer

**Affiliations:** 1 Graduate School of Education, Stanford University Graduate School of Education, 520 Galvez Mall, Stanford, CA 94305 USA; 2 Department of Psychology and Neuroscience, University of North Carolina at Chapel Hill, Chapel Hill, USA

**Keywords:** Human behaviour, Endocrinology, Risk factors

## Abstract

This longitudinal, within-subjects study examined whether adolescents’ biological sensitivity to socioeconomic status (SES) for emerging social difficulties varied day to day. Diverse adolescents (*N* = 315; ages 11–18; 57% female; 25% Asian, 18% Latinx, 11% Black) provided daily diaries and saliva samples for 4 days. We measured biological sensitivity as daily fluctuations in diurnal cortisol slope, and SES as a principal component of family income and maternal education. A robust analysis of 1013 daily assessments revealed that youth from lower SES homes reported greater social difficulties *only* on days that they exhibited flatter diurnal cortisol slopes, and youth from higher SES homes reported fewer social difficulties on these days. SES was not associated with social difficulties on days that adolescents exhibited steeper, declining diurnal cortisol slopes. Findings support recent theory that risk and resilience are dynamic processes that change within individuals over time. For better and for worse, youth may be more biologically sensitive to their family socioeconomic environments on days that their diurnal cortisol rhythms are flattened.

Adolescents raised in families with low socioeconomic status (SES) face heightened risk for experiencing environmental adversity and exhibiting developmental difficulties such as social conflict with peers, externalizing behaviors, and victimization^1,2^. However, adolescents are not always equally susceptible to socioeconomic disadvantage. The theories of Differential Susceptibility and Biological Sensitivity to Context suggest that levels of the stress-hormone cortisol identify differences in susceptibility between youth^3–5^, such that some youth are more sensitive to the effects of family socioeconomic environments than others^6^. In particular, youth who are sensitive to their environments struggle in environmental adversity (i.e., family poverty), but thrive in high-resourced and enriching environments^6^. For example, youth with heightened cortisol response display more optimal social functioning (e.g., fewer problematic social behaviors, more positive peer relationships) when they live in families with more socioeconomic resources, but more social difficulties when they live in families with fewer socioeconomic resources^7,8^. In contrast, children with lower cortisol response are less biologically sensitive, in that they exhibit similar levels of social adaptation across the range of family socioeconomic environments^7,8^. This research and other work^9^ suggests that individual differences in cortisol can identify youth who are particularly at risk or resilient to the effects of family-level socioeconomic adversity.

To date, prior research on biological sensitivity to context has focused on between-person analyses, which categorize youth into either a “sensitive” or a “non-sensitive” group^9^. Via this approach, biological sensitivity is measured as a one-time, trait-level characteristic that is assumed to be stable within individuals. Contrasting this prior empirical approach, researchers have recently theorized that biological sensitivity to context is probably not a one-time, constant trait that denotes individual vulnerability^10^. Instead, the adaptation-based approach to resilience^10^ underscores that risk and resilience are dynamic processes through which individuals respond and change to their environment over time. Specifically, whether an individual appears at-risk or resilient is not stable, and individuals’ biological sensitivity to their environments may change over time. Given this emergent theory that biological sensitivity changes over time, it is perhaps surprising that no known empirical studies have yet examined whether biological sensitivity to family socioeconomic context fluctuates over time within individuals. For example, are youth more sensitive to their family socioeconomic environment on some days compared to others? This inquiry requires repeated longitudinal measurements on multiple days and within-subject analyses that control for between-subject effects.

In addition, research to date on biological sensitivity has focused on salivary cortisol reactivity measured in response to laboratory stressors, which reflects acute increases in cortisol levels as an individual reacts to environmental stress or challenge^7,9,11,12^. However, it is also possible that naturally-occurring fluctuations in diurnal cortisol in daily life (i.e., outside the research laboratory) could mark biological sensitivity. Diurnal cortisol levels in the body fluctuate naturally throughout the day, typically peaking 30–45 min after waking, and declining from afternoon to evening in a rise-and-fall pattern^13^. The normative decrease in secreted cortisol from morning to evening is commonly known as the *diurnal cortisol slope*^13^. Despite this typical, healthy pattern, individuals vary in their daily cortisol rhythms from day to day, and contextual experiences impact cortisol release^13^. Moderately declining diurnal cortisol slopes are ideal and healthy, whereas blunted or flattened diurnal cortisol slopes reflect a sub-optimal or dysregulated cortisol rhythm that is linked with environmental stress and health, emotional and social difficulties^13^. Indeed, the diurnal cortisol slope is uniquely impactful for adolescents’ social functioning^13,14^. For instance, steeper, more declining diurnal slopes are associated with more optimal social adaptation (e.g., more positive peer relationships, fewer behavioral problems), whereas flatter slopes are associated with more social difficulties^13^. As such, diurnal cortisol slopes both partially reflect youths’ environmental experiences and also are closely tied to their social adjustment^15,16^. Since blunted (i.e., flattened) diurnal cortisol slopes are associated with both stress and suboptimal social development^13^, adolescents may be more biologically sensitive to their environments on days that they show blunted diurnal cortisol slopes.

This longitudinal, within-subjects study tests the novel hypothesis that adolescents’ biological sensitivity to SES for emerging social difficulties varies *within* individuals from day to day. We indexed daily biological sensitivity via daily fluctuations in diurnal cortisol slope. We hypothesized that a pattern of flattened cortisol throughout the day would mark daily biological sensitivity, such that adolescents would experience more social difficulties on days they had flattened cortisol slopes if they came from families with low SES, but fewer social difficulties on days they had flattened cortisol slopes if they came from families with high SES. The findings from this study will inform our understanding of human risk and resilience by illuminating whether biological sensitivity is a character-like trait that infers individual vulnerability, versus a *time-varying* process through which youth adapt and respond to their environment over time. If biological sensitivity is a time-varying characteristic, this offers hope that resilience is malleable and can inform efforts to support children’s positive development.

To test the hypothesis that biological sensitivity to family socioeconomic context changes over time, we drew on a sample of 315 racially and socioeconomically diverse adolescents who provided cortisol samples and daily diary measures each day for 4 days. We focused on adolescence, a key developmental transition period that involves heightened sensitivity to sociocultural contexts^17^, significant hormonal changes, and risk for social conflict with peers^18^. We collected four saliva samples each day across four days and focused on diurnal cortisol slope as a marker of diurnal cortisol functioning because it is particularly important for adolescents’ social adaptation^13^. We measured daily diary reports of social difficulties such as experiences of conflict, arguments, aggression, and victimization with peers. Parents reported on their family income and maternal education levels, which provided a multidimensional index of family SES. For analysis, we used linear mixed-effect models that nested days (Level 1) within participants (Level 2). We tested whether daily-level social difficulties varied as a function of the interaction between daily-level cortisol slopes and person-level SES. Our diverse sample allowed us to capture wide variability in both family income and maternal education. Further, this multi-informant approach minimized methodological bias. Our robust, multilevel analytical approach of 1013 daily assessments isolated the effects of both between- and within-subject biological sensitivity allowing us to test whether daily diurnal cortisol slope moderates the link between family SES and youths’ daily social difficulties.

## Results

Table 1 displays descriptive statistics and bivariate correlations. Adolescents from families with higher SES were younger, showed steeper (i.e., more negative) cortisol slopes, and reported fewer social difficulties. Younger adolescents showed flatter cortisol slopes, and girls reported more social difficulties compared to boys. There were no other significant correlations.

**Table 1:** Bivariate correlations between study constructs.

	Cortisol slope	Social difficulties	Family SES	Age
Cortisol slope	1			
Social difficulties	− 0.02	1		
Family SES	− 0.13*	− 0.16**	1	
Age	0.30***	0.060	− 0.28***	1
Girls	− 0.05	0.14**	− 0.01	0.07

*M*	− 1.27	0.05	− 0.25	14.63
*SD*	0.74	0.09	1.09	1.39
*Min*	− 4.51	0.00	− 2.58	11.91
*Max*	1.18	1.00	1.07	18.02

***p < 0.001, **p < 0.01, *p < 0.05. Values for time-varying variables (i.e., cortisol slope and social difficulties) are person-mean values averaged across all days within an individual adolescent. The sample was 57% female.

**Figure 1:**
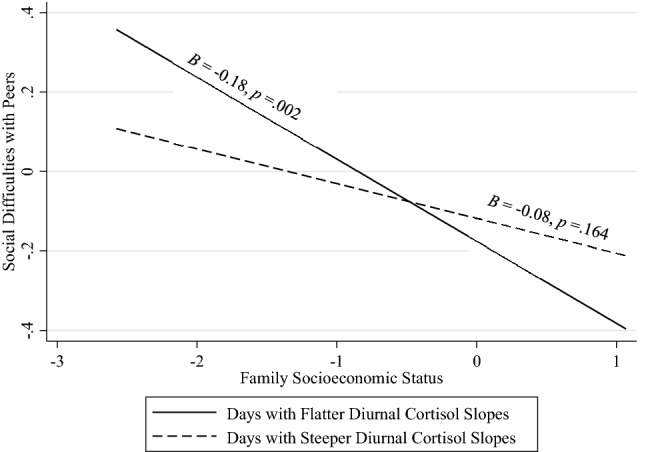
A negative association between family SES and social difficulties with peers emerged only on days that youth showed flatter diurnal cortisol slopes, a marker of daily biological sensitivity to context. In contrast, family SES was not related to social difficulties on days that youth showed steeper, declining diurnal cortisol slopes.

Table 2 displays standardized multilevel regression results. Model 1 demonstrates direct associations on daily and average levels. On average, youth with lower family SES reported more social difficulties. However, this direct association was qualified by significant cross-level interaction in Model 2. Specifically, the cross-level interaction between family SES and daily diurnal cortisol slope was significantly associated with social difficulties. As shown in Fig. 1, adolescents from lower SES reported more social difficulties only on days that they exhibited flatter cortisol slopes, whereas adolescents from higher SES reported fewer social difficulties on these days. In contrast, SES was not associated with social difficulties on days that adolescents exhibited steeper (i.e., more negative) cortisol slopes.

**Table 2:** Multilevel linear regression models.

	Social difficulties					
	Model 1		Model 2		Model 3	
	*β*	*SE*	*β*	*SE*	*β*	*SE*
School day	0.248***	(0.060)	0.242***	(0.060)	0.242***	(0.060)
Daily cortisol slope	0.027	(0.032)	− 0.037	(0.039)	− 0.037	(0.039)
Person average cortisol slope	− 0.031	(0.075)	− 0.030	(0.075)	− 0.007	(0.083)
Family SES-Education and income	− 0.147**	(0.050)	− 0.147**	(0.050)	− 0.092	(0.098)
Daily slope X SES			− 0.077**	(0.026)	− 0.077**	(0.026)
Average slope X SES					0.044	(0.068)
Constant	− 0.150	(0.115)	− 0.147	(0.115)	− 0.112	(0.126)
Observations	1013		1013		1013	
Number of groups	315		315		315	

Standard errors in parentheses. ***p < 0.001, **p < 0.01, *p < 0.05, Models additionally control for gender, age, and race/ethnicity (not shown).

Model 3 demonstrates that this cross-level interaction remained significant when controlling for average-level, between-subject interactions. These findings suggest that the association between SES and daily social difficulties varies by daily levels of diurnal cortisol within individuals, over and above between-person associations.

## Discussion

Across the social and biological sciences, there is increasing recognition that human risk and resilience are dynamic, context-specific processes that fluctuate across time, not stagnant biological traits that infer individual vulnerability^10,19^. This study models risk and resilience for social difficulties as a dynamic, evolving process that varies across days within individuals. Extending the theories of Differential Susceptibility^3^ and Biological Sensitivity to Context^4,5^, our study provides empirical evidence to support emergent theory that biological sensitivity to context is not a trait marker but rather a time-varying process which fluctuates within individuals from day to day. Specifically, our results suggest that adolescents are more susceptible to the adverse social effects of low family socioeconomic status (SES) on days that they exhibit relatively more flattened cortisol slopes that deviate from their own normative, healthy rise and fall in cortisol levels throughout the day.

Our study found that relatively flattened, diurnal cortisol slopes marked daily biological sensitivity to family SES. Specifically, youth from lower SES families reported more social difficulties (e.g., more conflict, aggression, social exclusion, and victimization with peers) on days that they exhibited flatter diurnal cortisol slopes, whereas youth from higher SES families reported fewer social difficulties on days that they exhibited flatter diurnal cortisol slopes. As such, a pattern of flattened cortisol slopes emerged as a marker of daily biological sensitivity to both adverse and enriching family socioeconomic contexts. Further, a pattern of relatively more steeply declining slopes into the evening emerged as a protective factor for social risk among youth experiencing socioeconomic hardship. Days on which adolescents exhibit flat diurnal slopes may reflect days when they are more sensitive to contextual influences—both positive and negative.

In households with low SES, flat diurnal slopes may reflect heightened sensitivity to stressors that youth from low SES homes disproportionately face, such as financial worries, neighborhood crime, household instability, or barriers to housing, education, health, or social services. Days on which youth exhibit flattened cortisol slopes may reflect “dual stress” that exacerbates the challenges associated with low SES and contributes to social difficulties with peers. Indeed, flatter cortisol slopes have been linked with both daily and prolonged stress^16^, which could explain why they serve as a dual risk factor in contexts of low SES. In contrast, a pattern of rise and fall in cortisol slopes throughout the day has been linked with more optimal mental and physical health^16^, so may be protective for low SES youth on a daily level. Specifically, declining cortisol slopes may reflect adolescents’ adaptive coping with significant poverty-related daily challenges and barriers that day, helping them to maintain positive social interactions with peers.

Interestingly, flattened diurnal cortisol slopes were also linked on a daily level to more positive social relationships among youth from high SES homes. This finding is somewhat surprising since flattened slopes have been previously associated emotional stress^16^. It is possible that relatively flatter slopes are adaptive and beneficial in contexts where resources are available. One prior study found that children from relatively wealthier families showed more optimal developmental outcomes if they had lower levels of hair cortisol^20^, underscoring that lower levels of physiological response can mark greater sensitivity^21,22^. Indeed, the specific biological profile marking greater sensitivity may differ by biological measure and timing^10^. Thus, while theory and empirical work indicate that some children are more or less biologically susceptible to contextual influences, our study converges with prior work to suggest that whether a pattern of low or high reactivity marks greater sensitivity depends on the biological marker and timing of measurement^10,19^. Our finding suggests that flatter slopes mark days when family resource are linked to adolescents’ social wellbeing “for better and for worse,” consistent with the differential susceptibility hypothesis^23^.

Our results extend the theories of Differential Susceptibility and Biological Sensitivity to Context in two more ways. First, prior research on biological sensitivity to context has focused on between-person analyses, representing biological sensitivity as a trait level characteristic that categorizes youth into “sensitive” or “non-sensitive” groups^4,23^. We extend this work by demonstrating that biological sensitivity varies across time within youth, and is not necessarily a stable or unchanging characteristic. Measuring human physiology across multiple time frames (e.g., multiple days) can illuminate an additional layer of sensitivity that is not reflected in one-time measures of physiological reactivity^24^. Supporting the notion that biological sensitivity varies from day to day—over and above between-subject differences—our results were robust to controls for trait-level biological sensitivity via average-level effects across days.

Second, our study demonstrates that cortisol marks biological sensitivity during adolescence, a unique period of both hormonal changes and social risk^18^. Prior research using cortisol as a marker of sensitivity has concentrated on early and middle childhood^9^. Finally, while prior studies measured biological sensitivity as reactivity to laboratory stressors, we demonstrate that naturally occurring diurnal cortisol levels can mark biological sensitivity. In sum, our findings contribute to a growing body of evidence that risk and resilience vary across a wide range of biological systems, timings, and measurements.

## Limitations and future directions

We acknowledge study limitations. We were unable to control for smoking, alcohol, or medication use which could impact cortisol^13^. Days that adolescents did not respond to the diaries or did not provide cortisol samples might represent the most difficult days and are missing from the analysis. Further, our measure of social difficulties was based on yes/no items. Future work should incorporate more detailed measures and investigate *why* lower slopes might be uniquely protective for social adaptation. Future research should also test other aspects of adolescents’ adaptation (e.g., emotional wellbeing, internalizing symptoms) and clarify whether our findings are generalizable in other cultural and socioeconomic contexts. Finally, our study was correlational and causality cannot be inferred. While we interpret our results with cortisol as the moderator of adolescents’ environments and their social adjustment, the direction of associations could be in the opposite direction. Specifically, it is possible that daily differences in social difficulties interact with SES and contribute to differences in cortisol slopes as a result of a combination of chronic and acute stressors.

Studying stress-physiology indexed via levels of the hormone cortisol provides unique insight into how humans respond to contextual experiences that are not readily observable or indicated by self-report^25^ but are meaningfully linked to development^13^. The theories of Differential Susceptibility and Biological Sensitivity to Context propose that particular physiological profiles can be maladaptive in contexts of environmental adversity, but healthy and promotive in contexts with sufficient resources^3,5,26^. However, as other researchers have suggested, stress physiology is a dynamic process of adaptation to environmental experiences, and not a one-time biological trait that denotes individual vulnerability^27^. Our study builds on prior research by revealing that youth are more susceptible to poverty-related stressors on days they exhibit flat diurnal cortisol slopes, a marker of daily biological sensitivity context to both adverse and enriching environments^16^. As such, our study provides empirical evidence to support the emergent theory that risk and resilience is a dynamic and time-varying process^10^—biological sensitivity may even vary across days within short periods of a single lifespan.

## Methods

### Sample and procedure

Participants were 315 adolescents (57.46% female; *M*_age_ = 14.63 years, *SD* = 1.39 years; range 11–18) who had at least one day of data for adolescents’ cortisol samples, adolescents’ daily diary reports of social difficulties, and parental report of SES, drawn from a larger sample of 370 adolescents. The analytical sample of 315 did not differ significantly from the larger sample of 370 in terms of demographic characteristics or levels of key study variables (*p*s > 0.13). The analytical sample was racially diverse: 40.3% were Non-Latinx White (N = 127), 25.4% Asian (N = 83), 14.6% Latinx (N = 45), 10.8% Black (N = 34), and 8.3% Other race (N = 26). Approximately 10% of mothers had less than an eighth-grade education, 13% did not complete high school, 24% completed high school, 27% completed postsecondary education, and 23% completed graduate school (3% declined to answer). Family/household income ranged from less than $14,999 to more than $90,000 (Median = $60,000–$74,999). Participants were recruited from the community using convenience sampling (e.g., posting flyers at schools and on listservs). Participants were compensated up to $20 for completing daily diaries and saliva samples and received a $20 bonus if inspection of the data indicated that they had completed all the diaries and saliva samples correctly and on time.

Participants were given daily diary checklists and saliva collection kits for each day of the study. The current study uses data from a larger study for which 14 days of daily diaries were collected. Participants provided cortisol samples on days 2–5 of the larger 14-day data collection period, so this study uses data only from days 2–5, because cortisol is a key focus variable. Most participants completed all days of their dairies (94.90%) and all of their saliva samples (98.92%) across the 4 days used for analysis. There were 1228 total person-day (i.e., Level 1) observations. Diaries included both weekdays and weekends. The order of days differed between participants depending on the day of the week that they started, but all participants had the same proportion of weekday to weekend data if they completed all of the diaries. Participants were instructed to complete their diary in the evening before bedtime either on paper (63.20%) or online (36.80%). Participants who responded with paper and pencil were given 14 manila envelopes and an electronic time stamper (Dymo Corporation, Stamford, CT), which verified the time that checklists were completed. Participants placed their completed checklists into a sealed envelope each night and stamped the seal of the envelope with the time stamper. Participants who completed surveys online were sent an email with the link to each daily diary survey, and the time and date of completion were recorded via the website. The daily diary checklists each took approximately 5–10 min to complete. All participants and legal guardians provided written informed consent/assent. All procedures were approved by the ethics committee at the University of Illinois at Urbana-Champaign Committee on Human Subjects (Protocol #13378; *Development of Decision Making and Social Cognition*) and carried out in accordance with regulations.

### Measures

#### Family SES

Family SES was calculated via principal components analysis of maternal education and household income. Specifically, mothers reported their education level, which ranged from 0 (representing less than 8th grade completed) to 6 (representing completed graduate school). Mothers also reported their household income.

#### Diurnal cortisol slope

Participants provided saliva at four time-points each of 4 days, for a total of 16 samples: (a) immediately upon waking up, (b) 30 min after waking up, (c) 5 p.m. (or before dinner), and (d) 8 p.m. (or before bed). Participants were instructed to take their samples before or > 30 min after brushing teeth, drinking, eating, or using tobacco. Raw cortisol values exceeding 60 nmol/L were flagged as outliers and excluded from analyses. Participants recorded the timing of each sample using a log-card and stamped with the electronic time stamper, which printed the current, unalterable, date and time. Participants stamped the card beside the heading for each sample and immediately placed the sample in their fridge. At the end of the saliva collection days, the samples were transferred to the research laboratory and stored in a − 80 °C freezer. At the end of the study, the samples were shipped to the Laboratory of Biological Psychology at the Technical University of Dresden, Germany where they were assayed using high-sensitivity chemiluminescence-immunoassays (IBL International, Hamburg, Germany). The inter-assay coefficient of variation was < 8%. We computed diurnal cortisol slope, using standard formulas^13^. Specifically, diurnal cortisol slope represents the decrease in secreted cortisol from morning to evening. We computed diurnal cortisol slope as the difference between the fourth (bedtime) cortisol sample and the first morning sample, divided by the time elapsed between these two samples^28^. A relatively healthy diurnal cortisol slope is typically a steep, negative decline, whereas a flatter (i.e., less negative) slope is associated with greater stress and cardiovascular risk^13^. For diurnal cortisol slope, 24.69% of the variance occurred between-subjects, with the remaining with-subjects.

Social difficulties was a measure based on social-relational theories of adolescent development^29^, and other self-reported measures of peer conflict^30^, including those from prior daily diary studies^31^. Items on the daily checklist asked participants to indicate whether they had engaged in different behaviors with peers each day. Specifically, *Social difficulties* were calculated as the mean of 11 daily diary items, such that higher scores indicate more social difficulties. Each item was coded as 0 = no, 1 = yes. These items were: “You hit, kicked, or shoved a peer”, “You threatened, insulted, or made fun of a peer”, “You said something mean behind a friend’s back”, “You excluded or left a friend out”, “You lied to a friend”, “Someone online or in a text message threatened, insulted or made fun of you”, “You argued with a friend”, “You argued with a boyfriend or girlfriend”, “You were excluded or left out by friends”, “A peer said something mean behind your back”, and “A peer threatened, insulted, or made fun of you” (Cronbach alpha = 0.71). To create the composite score at the daily level, we took the average of the items each day, and at the person-mean level, we calculated the average across all days of the standardized items for each participant. Adolescents experienced at least one social difficulty on 26.87% of days, and no social difficulties on 73.13% of days. For social difficulties, 65.02% of the variance occurred between-subjects, with the remaining with-subjects.

#### Covariates

We controlled for whether it was a school day or not^32^, and for adolescents’ self-reported age, gender, and race/ethnicity. Race was dummy coded within each race (i.e., Latinx = 1, not Latinx = 0) and categorized into five groups: Black, Asian, Latinx, White, and Other Race. Gender was dichotomous (Boys = 0, Girls = 1).

### Statistical analyses

Linear mixed-effect models nested days (Level 1) within participants (Level 2). We person-centered each Level 1 predictor and controlled for person-mean values of each predictor^33^ to isolate within-person associations from between-person associations^34^. Accordingly, in the tables, “daily” variables reflect person-mean-centered daily-level cortisol, whereas “average” variables reflect values averaged across days within each individual. To probe significant interactions, we used the simple slopes technique at 1*SD* above and below the mean value of the moderator^35^. Missing data ranged from 0 to 8.14%. Analyses were conducted in Stata (StataSE, Version 17).

## Data Availability

The data and syntax used in this study are available from the corresponding author upon request.
